# The impact of Shanghai’s comprehensive smoke-free legislation on hospital admission and mortality rates for chronic obstructive pulmonary disease: an interrupted time series analysis

**DOI:** 10.1093/ntr/ntag080

**Published:** 2026-04-10

**Authors:** Lihang Sun, Dan Qin, De Chen, Huiting Yu, Ying Shi, Yafei Hu, Jingrong Gao, Chenchen Xie, Xin Chen, Haiyin Wang

**Affiliations:** Department of Health Technology Assessment Research, Shanghai Health Development Research Center (Shanghai Medical information Center), Shanghai 201199, China; School of Pharmaceutical Sciences and Yunnan Provincial Key Laboratory of Pharmacology for Natural Products, Kunming Medical University, Kunming, Yunnan 650000, China; Department of Tobacco Control and Monitoring Evaluation, Shanghai Municipal Center for Health Promotion, Shanghai 200040, China; Department of Vital Statistics, Shanghai Municipal Center for Diesase Control and Prevention, Shanghai 200336, China; Department of Health Technology Assessment Research, Shanghai Health Development Research Center (Shanghai Medical information Center), Shanghai 201199, China; Department of Tobacco Control and Monitoring Evaluation, Shanghai Municipal Center for Health Promotion, Shanghai 200040, China; Department of Tobacco Control and Monitoring Evaluation, Shanghai Municipal Center for Health Promotion, Shanghai 200040, China; Department of Tobacco Control and Monitoring Evaluation, Shanghai Municipal Center for Health Promotion, Shanghai 200040, China; Department of Vital Statistics, Shanghai Municipal Center for Diesase Control and Prevention, Shanghai 200336, China; Department of Health Technology Assessment Research, Shanghai Health Development Research Center (Shanghai Medical information Center), Shanghai 201199, China

**Keywords:** chronic obstructive pulmonary disease, tobacco control, tobacco-related disease, smoking/harm reduction, smoke-free legislation

## Abstract

**Introduction:**

Smoking and secondhand smoke pose major global health risks, contributing to chronic obstructive pulmonary disease (COPD). Evidence on the health impact of Shanghai’s smoke-free legislation is limited. This study assesses the effects of Shanghai’s smoke-free legislation on COPD hospital admission and mortality rates.

**Methods:**

Chronic obstructive pulmonary disease hospital admissions and deaths from July 2013 to December 2019 were analyzed using interrupted time series. Adjustments were made for demographics, weather, PM2.5, and seasonality.

**Results:**

A total of 358 941 COPD hospital admissions and 56 824 deaths were recorded. After implementation of the legislation, there was no evidence of a short-term reduction in either COPD hospital admissions or mortality among adults aged ≥35 years. Both COPD hospital admissions and mortality showed downward trends over the longer term, although these overall changes were not statistically significant. Subgroup analysis revealed a modest but statistically significant long-term decline in admissions among adults aged 35–64 years (β = −0.06, *P*<.05), while no statistically significant short-term mortality change was observed among adults aged ≥65 years. Age-standardized analyses and Poisson and negative binomial sensitivity models yielded similar admission trends and broadly consistent long-term mortality patterns.

**Conclusions:**

The implementation of the legislation was associated with a long-term downward trend in COPD-related hospital admissions and mortality, but the overall impact to date appears to be limited. Sustained implementation of stringent tobacco control policies is therefore recommended, alongside further improvement and expansion of existing measures to strengthen enforcement and broaden interventions, especially for outdoor smoking, to achieve greater health benefits.

**Implications:**

This study provides empirical evidence that Shanghai’s comprehensive smoke-free legislation was not associated with a detectable short-term reduction in either COPD hospital admissions or mortality. However, both outcomes showed downward trends over the longer term, with reductions in hospital admissions appearing more pronounced among older adults, underscoring that the health benefits of smoke-free policies may accrue gradually through sustained implementation. Strengthening enforcement, expanding smoke-free areas, and integrating complementary public health interventions—such as smoking cessation support and stricter outdoor smoking regulations—could enhance the long-term effectiveness of tobacco control policies. These findings highlight the need for continuous evaluation and policy refinement to maximize public health benefits and reduce the overall burden of COPD in urban populations.

## Introduction

According to WHO estimates, approximately 1 billion people worldwide, including 650 million children, are exposed to secondhand smoke, primarily due to smokers in their households or workplaces.^1^ Secondhand smoke causes approximately 1.2 million deaths annually, linked to cardiovascular diseases, lung cancer, and respiratory disorders.^2^ To reduce tobacco use and its associated health risks, WHO adopted the Framework Convention on Tobacco Control in 2003.^3^ Since its implementation, the Framework Convention on Tobacco Control has been signed and ratified by over 180 countries, covering most of the global population,^4^ and reducing the smoking prevalence among individuals aged 15 years and older to 19.2%.^5^ Despite these significant global achievements, progress varies considerably across countries.^6^^,^^7^

China is the world’s largest producer and consumer of tobacco, with over 300 million smokers, 90% of whom are men.^8^ In 2010, approximately 1 million deaths in China were attributed to smoking, a number projected to double to 2 million annually by 2030.^9^ Compared to efforts in monitoring, mass media campaigns, and taxation under the MPOWER tobacco control strategy, China scores the lowest in protecting the public from tobacco harm through smoking bans in public places.^10^ Following China’s ratification of the Framework Convention on Tobacco Control in 2005, tobacco control measures have steadily improved, though a unified national smoke-free law remains absent. Since 2008, more than 20 cities have enacted local smoke-free laws or regulations, prohibiting smoking in public spaces.^11^

Shanghai is a leading city in tobacco control in China. On March 1, 2010, the *Regulations of Shanghai Municipality on Smoking Control in Public Places* (hereafter referred to as the Regulations) came into effect. This was the first provincial-level tobacco control law enacted in mainland China following the Framework Convention on Tobacco Control ratification. The Regulations prohibit smoking in public places, including kindergartens, primary and secondary schools, maternity and child healthcare institutions, children’s hospitals, sports venues, publicly accessible cultural heritage sites, and public transportation waiting areas.^12^ After two revisions in 2016 and 2022, the Regulations now fully ban indoor smoking (including indoor public places, indoor workplaces, and public transportation) and include e-cigarettes in smoke-free public areas.^13^ Since the first revision of the Regulations, which implemented a comprehensive indoor smoking ban, the average annual penalties for smoking violations in Shanghai have exceeded 2.2 million CNY, with a cumulative total of nearly 16 million CNY by the end of 2023. The smoking violation rate in smoke-free venues has dropped from 37.5% before legislation to 12.3%, and the use of e-cigarettes in such venues is 2.2%.^14^ The adult smoking rate has decreased gradually, from 26.9% in 2010 to 19.42% in 2023.^15^ However, there is a lack of evidence evaluating the health impacts of the Regulations on Shanghai residents, highlighting the need for further research.

Smoking and secondhand smoke are well-established major risk factors for various chronic non-communicable diseases globally.^16^ Chronic obstructive pulmonary disease (COPD) is one of the most common smoking-related diseases. Long-term smoking damages lung tissue, impairs the respiratory system’s self-repair and defense mechanisms, and leads to irreversible breathing difficulties or even death.^17^ COPD is the third leading cause of death in China.^18^ According to the Global Burden of Disease 2021 report,^19^ smoking-related COPD deaths account for more than 40% of all COPD deaths worldwide. In China, 1–1.1 million COPD deaths occur annually, with 60%–70% linked to smoking. There is some evidence^20–25^ that smoke-free legislation reduces hospital admissions and deaths from COPD, but the results are heterogeneous according to economic conditions, the strength of policy implementation, and ethnic differences.

This study evaluates the impact of the Regulations on trends in COPD hospital admissions and mortality among the Shanghai registered population. It aims to provide local empirical evidence for the further optimization of tobacco control policies and the improvement of public health services in Shanghai.

## Methods

### Study design

This study used the Interrupted Time Series (ITS) methodology to evaluate intervention effects. ITS is a commonly used approach in public health, public policy, and health services research. It assesses the effectiveness of interventions by measuring immediate changes in the outcome variable’s level and slope alterations in regression lines before and after policy implementation.^26^

The intervention policy evaluated in this study was the first revision of the Regulations, which was implemented on March 1, 2017. The health outcomes examined were the monthly hospital admission and mortality rates of COPD among the Shanghai registered population. The study period spanned from July 1, 2013 to December 31, 2019. Potential confounders were considered, including seasonal effects, meteorological factors, and air pollutant concentrations, which may influence hospital admission and mortality rates.

### Data collection

Hospital admissions data for Shanghai registered population were obtained from the Shanghai Municipal Health Commission Information Center. COPD cases were identified using the International Classification of Diseases, 10th Revision codes J40–J44 and J47. To avoid overcounting, patients readmitted for the same condition within 28 days were excluded, while readmissions after 28 days were considered new cases. Mortality data were sourced from the all-cause mortality surveillance system managed by the Shanghai Center for Disease Control and Prevention, with COPD (J40–J44, J47) as the underlying cause of death.

Monthly meteorological data, such as average temperature and relative humidity, were obtained from the Shanghai Meteorological Bureau. PM2.5 concentration data were retrieved from a near-real-time air pollutant concentration database,^27^ a method widely applied in previous studies. Data on the registered population were derived from the Shanghai Statistical Yearbook.^28^ To estimate monthly population figures, the average of the year-end registered populations from 2 consecutive years was calculated.

### Statistical analysis

This study used three analytical methods to assess the short- and long-term impacts of the Regulations on monthly COPD hospital admission and mortality rates among the Shanghai registered population.

First, ITS analyses were conducted using an autoregressive integrated moving average model and an ordinary least squares regression model.^29^ Preliminary data analysis revealed minimal COPD hospital admission and mortality events among individuals under 35 years old, so the analysis was limited to individuals aged 35 years and older. The primary outcomes were the monthly COPD hospital admission and mortality rates (per 100 000 population), calculated by dividing the number of monthly hospital admissions or deaths by the estimated monthly population. A dummy variable defined policy implementation, with pre-intervention months coded as 0 and post-intervention months coded as 1. The models also included an interaction term between policy implementation and time to estimate changes in long-term trends. The policy intervention point was set at March 1, 2017, and the study period covered 79 months (44 months pre-intervention and 35 months post-intervention). The regression model formula was as follows:


$$\mathrm{Y}=\mathrm{\beta} 0+\mathrm{\beta} 1\mathrm{Tt}+\mathrm{\beta} 2\mathrm{Xt}+\mathrm{\beta} 3\mathrm{XtTt}+\mathrm{\varepsilon} \mathrm{t}$$

where Y represents the outcome variable (monthly hospital admission or mortality rates), Tt is the time since the start of the study, Xt is a dummy variable indicating policy implementation (0 for pre-intervention, 1 for post-intervention), β0 denotes the baseline hospital admission or mortality rate at the start of the study, β1 represents the pre-intervention trend, β2 captures the immediate changes in the outcome post-intervention, and β3 reflects the changes between the slopes of the outcome variable between the pre-intervention and post-intervention periods (a long-term effect).

Second, subgroup analyses stratified by age (35–64 years and ≥ 65 years) were conducted to explore the differential impact of the Regulations. Age 65 was chosen based on the United Nations’ aging classification.^30^ Given the increasing population aging trend in China from 2013 to 2019, and to avoid biasing the study results by directly comparing crude hospital admission and crude mortality rates, we used standardized methods^31^ to account for demographic differences across years. Using the 2013 Shanghai registered population as the reference population, age-standardized monthly COPD hospital admission and mortality rates were calculated for subsequent years.

Finally, sensitivity analyses were performed using ITS models based on Negative Binomial and Poisson distributions. Model fit was assessed using the Akaike Information Criterion and Bayesian Information Criterion.^32^ In these models, monthly COPD hospital admissions and deaths were outcome variables, with the annual registered population set as an offset variable (fixed coefficient = 1). Fourier terms (sine and cosine)^33^ were included to model seasonality, and adjustments were made for confounding factors,^34^ such as monthly average temperature, relative humidity, and PM2.5 concentrations.

A significance threshold of *P*<.05 was applied to determine statistical significance. All analyses were conducted using Stata v13, employing the “itsa” package and generalized linear models functions for statistical modeling.

### Patient and public involvement

This study utilized aggregated data from the Shanghai Municipal Health Commission and the Shanghai Center for Disease Control and Prevention. As the outcome indicators were based on monthly aggregated data, patients and the public were not directly involved in study design, data analysis, or interpretation of results.

## Results

### Basic information

During the study period, a total of 358 941 COPD patients were hospitalized, and 56 824 deaths were attributed to COPD (Table 1). The disease burden was concentrated in adults aged ≥65 years, who accounted for 310 376 admissions (86.47%) and 54 804 deaths (96.45%).

**Table 1:** Descriptive characteristics of the registered population with COPD hospital admissions and mortality in Shanghai from 2013.7 to 2019.12 (by age and year subgroups).

	**Hospital admissions**		**Mortality**	
	**Number of admissions**	**Annual admission rates (rate of change^a^)**	**Number of mortality**	**Annual mortality rates (rate of change^a^)**
**Age**				
** ≥35 years**	358 941	NA	56 824	NA
** 35–64 years**	48 565	NA	2020	NA
** ≥65 years**	310 376	NA	54 804	NA
**Year**				
** 2013**	44 362	462.58^b^	8190	85.40^b^
** 2014**	50 089	515.21 (11.38%)	9218	94.82 (11.03%)
** 2015**	54 252	551.21 (6.99%)	9655	98.10 (3.46%)
** 2016**	50 129	462.58 (−16.08%)	8867	88.87 (−9.41%)
** 2017**	54 516	536.80 (16.05%)	8454	83.24 (−6.33%)
** 2018**	52 868	512.08 (−4.6%)	8289	80.29 (−3.55%)
** 2019**	52 725	508.82 (−0.64%)	8246	79.58 (−0.89%)

^a^Rate of change in hospital admissions and mortality over the previous year.

^b^Adjusted to annual admission rate and annual mortality rate based on half-yearly hospital admissions and mortality data.

Abbreviations: COPD, chronic obstructive pulmonary disease; NA, not available.

In 2013, the annual COPD admission and mortality rates were 462.58 and 85.40 per 100 000 population, respectively. Both rates increased during 2014-2015, with admissions peaking at 551.21 per 100 000 in 2015 (a 6.99% increase from 2014) and mortality at 98.10 per 100 000 (up 3.46%). In 2016, both indicators declined, with the admission rate falling to 462.58 per 100 000 (−16.08%) and mortality to 88.87 per 100 000 (−9.41%).

Following approval of the amended smoke-free Regulation in November 2016 and its implementation in March 2017, COPD mortality showed a sustained downward trend from 2016 onward: rates were 83.24, 80.29, and 79.58 per 100 000 in 2017-2019, with annual changes of −6.33%, −3.55%, and −0.89%, respectively. The admission rate increased transiently in 2017 to 536.80 per 100 000 (up 16.05%), then decreased slightly in 2018-2019 to 512.08 and 508.82 per 100 000 (−4.60% and −0.64%).

### Hospital admission rates

#### Baseline and standardized results

**Figure 1:**
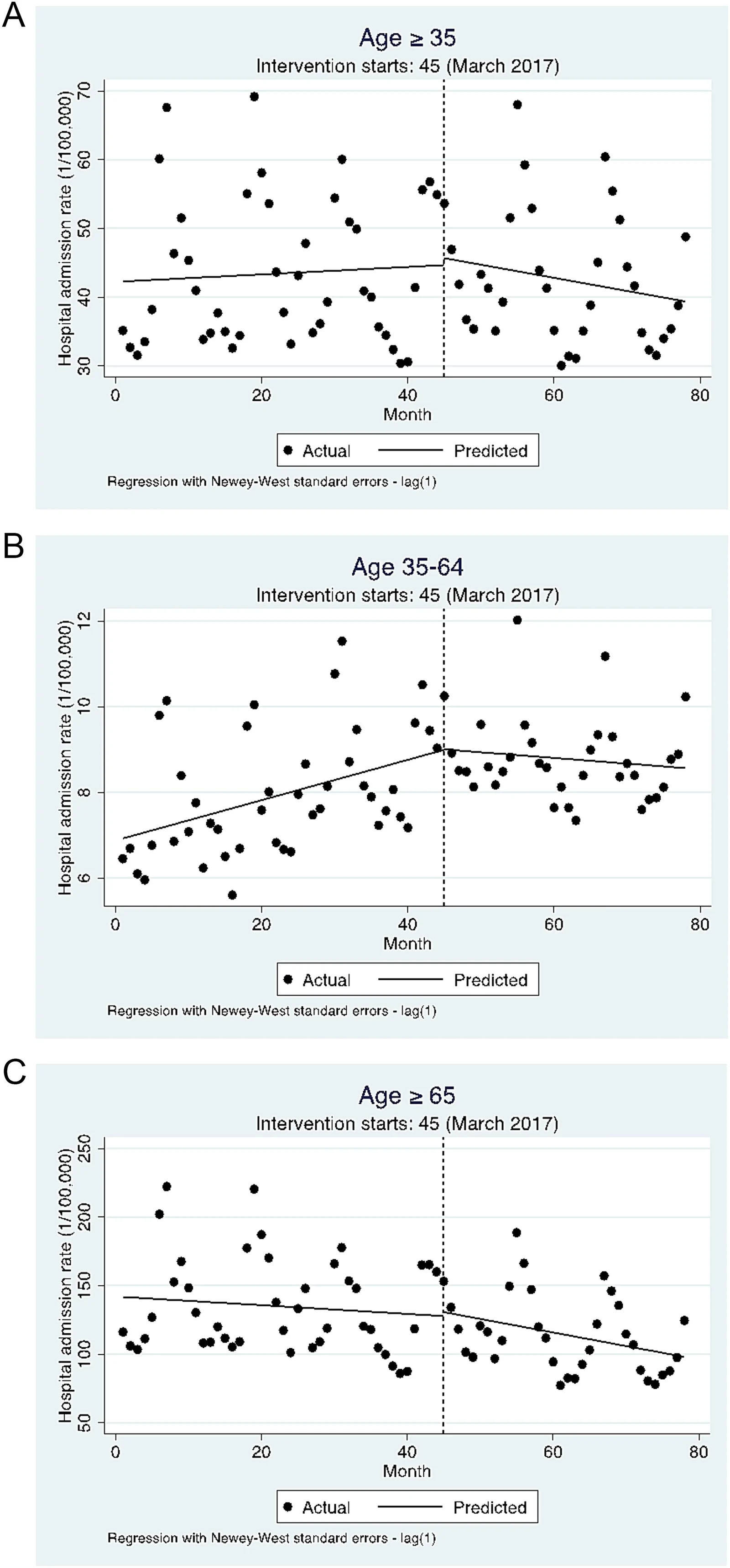
Monthly COPD hospital admission rates by age group in Shanghai, July 2013-December 2019. (A) Adults ≥35 years; (B) 35–64 years; (C) ≥65 years. COPD, chronic obstructive pulmonary disease.

After the intervention, the monthly COPD admission rate per 100 000 among adults aged ≥35 years showed a small, non-significant short-term increase (β = 1.01, *P*>*.*05), but a long-term declining trend (β = −0.25, *P*>.05), corresponding to about 304 fewer admissions per year (Figure 1, Table 2). By age group, the short-term effect in adults aged 35–64 years was close to zero (β = 0.01, *P*>.05), whereas the long-term admission rate declined significantly (β = −0.06, *P*<.05), preventing around 50 admissions annually. Among adults aged ≥65 years, the short-term increase in admissions was larger (β = 2.88, *P*>.05), but the long-term decline was also more pronounced (β = −0.67, *P*>.05), corresponding to about 247 fewer admissions per year. Age-standardization using the 2013 Shanghai population structure yielded trends similar to the crude results, with the long-term decline in admissions for those aged 35–64 years remaining significant (β = −0.05, *P*<.05).

**Table 2:** Baseline results, standardized results, and sensitivity analysis results of COPD hospital admission rates and mortality rates after the implementation of the amendments to the Regulations in Shanghai.

	**Baseline results**			**Demographic standardization**		**Poisson**		**Negative binomial**	
**Age**	**Level**	**Slope**	**Number of admissions/mortality avoided per year**	**Level**	**Slope**	**Immediate effect** **RR (95% CI)**	**Long-term effect** **RR (95% CI)**	**Immediate effect** **RR (95% CI)**	**Long-term effect** **RR (95% CI)**
**Hospital admission rates**									
** Over 35**	1.01	−0.25	304	1.01	−0.25	1.082^*^ (1.068-1.097)	0.995^*^ (0.994-0.996)	1.077 (0.103-2.051)	0.995 (0.952-1.038)
** 35–64**	0.01	−0.06^*^	50	0.01	−0.05^*^	1.043^*^ (1.005-1.081)	0.991^*^ (0.989-0.993)	1.042 (0.101-1.982)	0.991 (0.949-1.034)
** Over 65**	2.88	−0.67	247	0.75	−0.18	1.094^*^ (1.078-1.110)	0.994^*^(0.994-0.995)	1.088 (0.104-2.073)	0.994 (0.952-1.037)
**Mortality rates**									
** Over 35**	−0.43	−0.03	36	−0.43	−0.03	1.008 (0.974-1.042)	0.997^*^ (0.996-0.999)	0.987 (0.094-1.879)	0.997 (0.954-1.040)
** 35–64**	0.01	−0.003	2	0.01	−0.002	1.086 (0.896-1.277)	0.992 (0.983-1.000)	1.060 (0.072-2.048)	0.991 (0.947-1.036)
** Over 65**	−1.53	−0.07	25	−0.40	−0.02	1.011 (0.976-1.045)	0.996^*^ (0.995-0.998)	0.989 (0.094-1.884)	0.996 (0.953-1.039)

^*^ *P*<.05

Abbreviation: COPD, chronic obstructive pulmonary disease.

#### Sensitivity analysis results

After adjusting for seasonality and meteorological factors, Poisson and negative binomial models produced overall admission trends consistent with the main analysis (Table 2). In the Poisson model, the monthly admission rate among adults ≥35 years increased by 8.2% in the short term (RR = 1.082, 95% CI, 1.068-1.097), but showed a statistically significant 0.5% long-term reduction (RR = 0.995, 95% CI, 0.994-0.996). Among adults aged 35–64 years, admissions increased by 4.3% in the short term (RR = 1.043, 95% CI, 1.005-1.081) but declined by 0.9% in the long term (RR = 0.991, 95% CI, 0.989-0.993). Among those aged ≥65 years, admissions rose by 9.4% immediately after the policy (RR = 1.094, 95% CI, 1.078-1.110), followed by a modest long-term reduction of 0.6% (RR = 0.994, 95% CI, 0.994-0.995). In the negative binomial model, short- and long-term effects by age group were similar in direction and magnitude to the Poisson estimates but did not reach statistical significance. The negative binomial model had lower Akaike Information Criterion and Bayesian Information Criterion values than the Poisson model, indicating better model fit.

### Mortality rates

#### Baseline and standardized results

**Figure 2:**
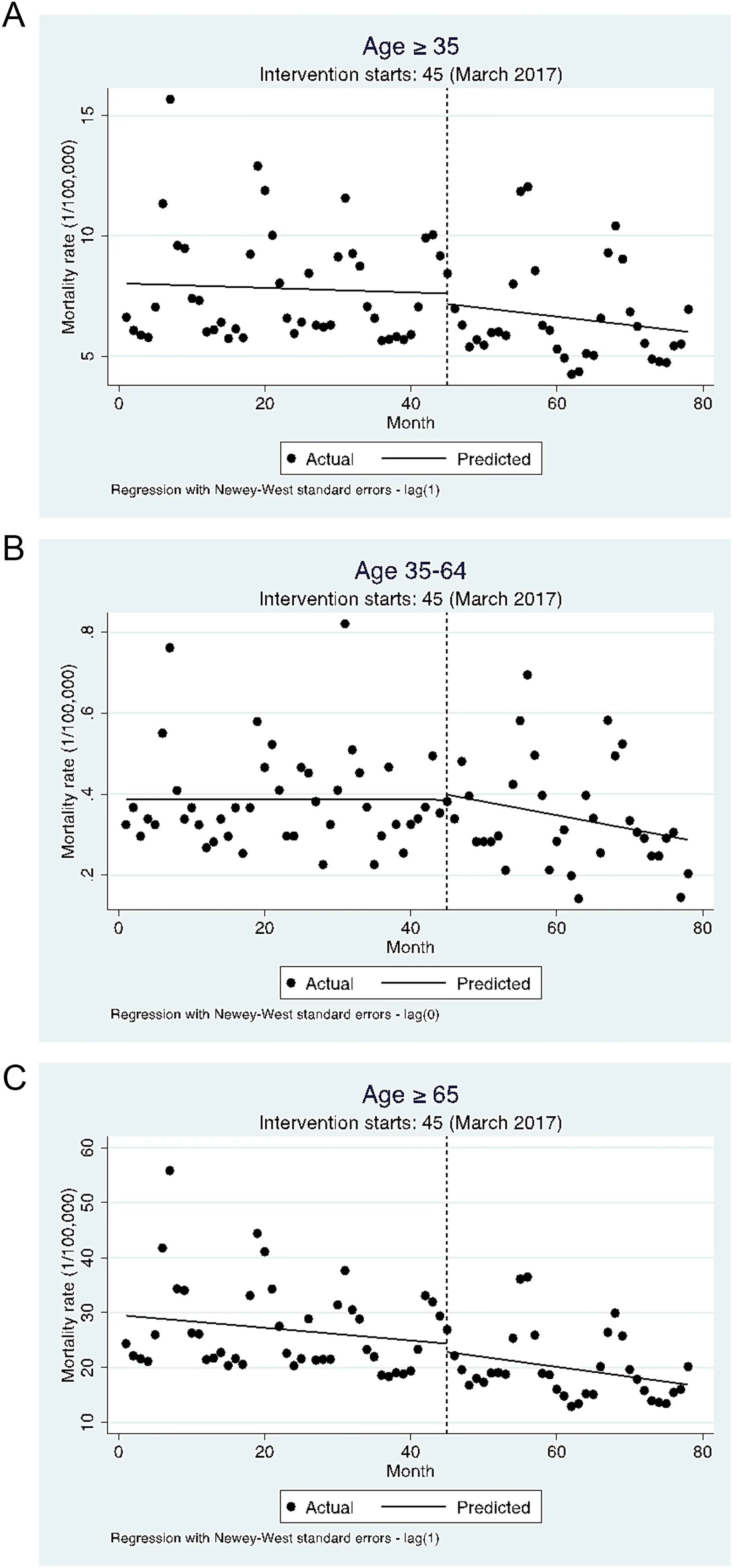
Monthly COPD mortality rates by age group in Shanghai, July 2013-December 2019. (A) Adults ≥35 years; (B) 35–64 years; (C) ≥65 years. COPD, chronic obstructive pulmonary disease.

After the intervention, the monthly COPD mortality rate among adults aged ≥35 years showed a small short-term decrease (β = −0.43*, P*>.05) and a slight long-term decline (β = −0.03, *P*>.05), corresponding to about 36 fewer deaths per year (Figure 2, Table 2). In adults aged 35–64 years, mortality increased slightly in the short term (β = 0.01, *P*>.05) and showed a very small long-term decrease (β = −0.003, *P*>.05), equivalent to around two fewer deaths per year. Among those aged ≥65 years, both short-term (β = −1.53, *P*>.05) and long-term (β = −0.07, *P*>.05) declines were larger in magnitude, with roughly 25 fewer deaths per year. Age-standardized analyses using the 2013 Shanghai population structure showed similar non-significant trends (Table 2).

#### Sensitivity analysis results

After adjusting for confounders, Poisson and negative binomial models yielded long-term mortality trends broadly consistent with the baseline results (Table 2). In the Poisson model, adults aged 35–64 years showed a non-significant short-term increase in admissions of 8.6% (RR = 1.086, 95% CI, 0.896-1.277) but a small, statistically significant long-term reduction in mortality (RR = 0.992, 95% CI, 0.983-1.000). For adults aged ≥35 and ≥65 years, short-term effects changed from a decrease in the baseline analysis to a slight increase, whereas long-term effects remained in the same direction and were significantly reduced (≥35 years: RR = 0.997, 95% CI, 0.996-0.999; ≥65 years: RR = 0.996, 95% CI, 0.995-0.998). In the negative binomial model, short- and long-term effects across age groups were in the same direction as the baseline estimates, with decreasing trends for adults aged ≥35 and ≥ 65 years, but none reached statistical significance.

## Discussion

This is the first study in Shanghai to use an interrupted time-series design to assess the health impact of smoke-free legislation on COPD. Between July 2013 and December 2019, there were 358 941 COPD hospitalizations and 56 824 COPD deaths. After implementation of the comprehensive indoor smoking ban, the admission rate among adults aged ≥35 years showed a slight short-term increase but a long-term declining trend (β = −0.25, *P*>.05), corresponding to about 304 fewer admissions per year. Mortality also decreased in the short and long term, but the effects were small and not statistically significant. Age-standardized and covariate-adjusted sensitivity analyses showed similar trends to the baseline models, although the statistical significance differed between the Poisson and negative binomial specifications.

Previous studies have assessed the health impacts of smoking control policies on COPD patients, but their findings are heterogeneous due to differences in economic conditions, policy enforcement, population demographics, and model selection. Even cities implementing similar policies show variations. In contrast to the short-term admission rate trends observed in this study, Beijing tobacco control policy^20^ led to an immediate 14.7% reduction in weekly COPD admission rates. This achievement was attributed to Beijing stringent enforcement measures, including fines and extensive public education campaigns, which yielded rapid short-term effects.^35^ The Shanghai Regulations, effective since 2010, already restricted indoor smoking in most public places, except a few like restaurants. Therefore, health effects may have been observed earlier, and the impact of the 2017 amendment, which introduced a full indoor smoking ban, was less pronounced.^36^ Additionally, Beijing saw a decline in long-term hospitalization rates (−3.0%), with 5581 fewer hospital admissions over 25 months following the policy’s implementation. Similarly, our study observed a long-term downward trend in COPD admissions in Shanghai (β = −0.25, *P*>.05), equivalent to approximately 304 fewer admissions per year. The consistent long-term declining direction observed in both cities suggests that the health benefits of tobacco control policies may accumulate gradually over time. In Beijing, the long-term reduction was smaller relative to the pronounced short-term decline, indicating that the initial policy shock produced the most immediate impact. In contrast, Shanghai showed no clear short-term effect but a gradual long-term decline, which may reflect the progressive strengthening of policy enforcement and compliance over time.

In Switzerland,^21^ COPD hospital admissions rates across 14 regions with the highest COPD burdens declined by 2.08% over the long term, although the reduction varied significantly based on economic development levels. For instance, the Graubünden region exhibited a more pronounced decline in hospital admissions (−22.4%) compared to other areas (−7.0%).^21^ Similarly, a Spanish study^23^ reported substantial reductions in COPD-related hospital admissions (−16.6% to −20.1%) in economically disadvantaged areas. However, disparities were also observed in economically developed regions: no significant change was detected in Madrid, whereas Barcelona saw a 16.0% reduction in COPD hospital admissions.^24^ It should be noted that the international disease codes selected by the Swiss and Spanish studies differ slightly from ours. Furthermore, factors such as seasonal variations, temperature, influenza epidemics, and acute respiratory infection rates, which were controlled in the Spanish study, may contribute to differences in findings.

Unlike the Shanghai findings, an Irish study^25^ reported substantial immediate reductions (26%–51%) in monthly COPD mortality rates across all age groups (35–64, 65–84, and ≥85 years), particularly among those aged ≥85 years (RR: 0.49; 95% CI, 0.46–0.83). However, the long-term effects in Ireland were less pronounced, while Switzerland^20^ reported a 13.9% reduction in long-term COPD mortality rates. Differences in genetic susceptibility, smoking intensity, smoking methods, and environmental or occupational exposures might account for the higher COPD incidence and mortality rates among European populations compared to Asian populations.^37^

In our study, the modest decline in COPD mortality rates in Shanghai may be attributed to several factors. First, the mortality rate of COPD typically shows a time-lag effect. The long-term impact of smoking may take decades to become evident.^38^ Although the smoking rate in Shanghai has decreased, improvements in mortality may still take a longer time.^39^ Second, despite continuous advancements in medical techniques and quality, such as new therapeutic options and pulmonary rehabilitation, COPD remains an incurable disease and is often misdiagnosed or undiagnosed due to the subtlety of early symptoms, contributing to its persistently high mortality rate.^40^^,^^41^ Additionally, the mortality rate of COPD is influenced by comorbidities, such as cardiovascular disease and lung cancer.^42^ If management of these conditions does not improve simultaneously, the decline in COPD mortality may be slower. Finally, as a megacity with a large population, Shanghai has seen a significant reduction in smoking rates, yet over 4 million people still smoke. While the implementation of the Regulations has led to positive outcomes in indoor smoke-free environments, outdoor smoking and the resulting second-hand smoke exposure remain a concern.^43^

Our findings provide evidence of differential intervention effects across populations. The 2017 comprehensive indoor smoking ban was more effective in reducing COPD admissions and mortality among adults aged ≥65 years than among those <65 years. This observation aligns with findings from Beijing,^20^ Switzerland,^21^^,^^22^ Spain,^23^^,^^24^ and Ireland.^25^ The 2017 revision of Shanghai’s Tobacco Control Regulations bans smoking in indoor public places, workplaces, and public transportation, as well as in outdoor areas like crowded public transport waiting zones, which are key settings for older adults and may partly explain the larger effects observed in this age group. Older adults also have a much higher baseline risk of developing and dying from COPD. As a result, even small relative reductions in risk in this group can lead to a larger absolute decrease in the number of admissions and deaths.

In sensitivity analyses, Poisson and negative binomial models produced trends broadly consistent with the baseline results. However, in the Poisson models adjusted for confounders among adults aged ≥35 and ≥65 years, the short-term mortality effect reversed from a small decline in the baseline models to a slight increase, indicating that the apparent immediate drop was largely explained by seasonal, meteorological, and air-pollution fluctuations, whereas the long-term downward trend in mortality was more robust to covariate adjustment. In addition, the negative binomial models, which had better fit based on lower Akaike Information Criterion and Bayesian Information Criterion values(Table S1-S2), indicated no statistically significant short- or long-term changes in COPD hospital admission and mortality rates. In contrast, the Poisson model produced statistically significant results. This discrepancy likely reflects the different assumptions about dispersion: negative binomial models are more sensitive to overdispersion,^44^ while in our data, the monthly counts were relatively concentrated, so the Poisson models may have produced smaller SEs and thus statistically significant results.^45^

### Strengths and limitations

This study has several strengths. First, the data were sourced from official government sources, encompassing a large and relatively closed population, thereby minimizing selection bias and enhancing internal validity. The extended 79-month study period effectively captured the policy’s long-term impacts, reducing random effects and improving model fit. Second, we rigorously adhered to the International Classification of Diseases, 10th Revision classification system, incorporating data on COPD-related conditions (J40–J44, J47). Third, standardized hospital admission and mortality rates were used to account for aging trends and age-structure differences, providing robust and unbiased evidence of policy effects. Lastly, we controlled for confounders, including seasonal variations, meteorological conditions, and air pollution, ensuring the accuracy and robustness of results.

However, this study has limitations. First, we did not include geographical, population-based, or non-smoking-related disease control groups. We initially tried to use outcomes unrelated to smoking as negative controls. However, their baseline levels and pre-intervention trends were very different from those of COPD, which broke the comparability assumption and could have introduced bias, so we did not retain them as controls. Although ITS analysis does not necessarily require a control group, incorporating one could exclude the potential influence of background changes, other policies, or events.^46^ Future studies should include appropriate control groups to enhance robustness. Second, due to data availability, we could not conduct sex-specific subgroup analyses within age groups. The 2021 data indicate significant gender differences in smoking rates (36.8% for males and 0.8% for females),^47^ highlighting the need for improved databases to facilitate gender-specific analyses. Third, although we adjusted for meteorological factors and PM2.5, we were unable to include other pollutants such as NO₂ and O_3_. As this is an ecological study, we also could not account for individual-level confounders such as smoking status, education, tobacco affordability, or the prevalence of obesity, influenza, and other respiratory infections, which may have introduced bias into our findings. Lastly, while the implementation of the regulation and strict enforcement has significantly reduced smoking rates, outdoor secondhand smoke exposure may dilute the overall health effects.^43^ It is recommended to further strengthen outdoor smoking control and advocacy efforts, building on the foundation of comprehensive indoor smoking bans, with the aim of further tracking and evaluating the impact of tobacco control policies in the future.

## Conclusion

This study provides valuable evidence on the health benefits of the *Regulations of Shanghai Municipality on Smoking Control in Public Places*. The implementation of the Regulations was associated with a long-term downward trend in COPD hospital admissions and mortality. These findings suggest that smoke-free policies can contribute to improving respiratory health, but that the overall impact to date has been limited. Building on the existing indoor smoking ban, Shanghai and the rest of the country should further strengthen tobacco control policies, enhance enforcement, and better manage outdoor smoking in order to achieve greater population health benefits.

## Supplementary Material

ntag080_Supplementary_material_and_tables

## Data Availability

Data are available upon reasonable request. Hospital admissions data for Shanghai registered population were obtained from the Shanghai Municipal Health Commission Information Center. Mortality data were obtained from the Shanghai Center for Disease Control and Prevention. Monthly meteorological data were obtained from the Shanghai Meteorological Bureau (http://sh.cma.gov.cn/sh/tqyb/wrtqyb/). PM2.5 concentration data were retrieved from a near-real-time air pollutant concentration database (http://tapdata.org.cn/). Data on the registered population were derived from the Shanghai Statistical Yearbook (https://tjj.sh.gov.cn/tjnj/index.html).
