# Supplementary material for: The impact of Shanghai’s comprehensive smoke-free legislation on hospital admission and mortality rates for chronic obstructive pulmonary disease: an interrupted time series analysis
Source: Nicotine Tob Res. 2026 Apr 10;28(9):1555–63. doi: 10.1093/ntr/ntag080 (PMC13500350; doi:10.1093/ntr/ntag080)
Supplement: Supplementary file 1 [file ntag080_supplementary_material_and_tables.docx]

**Supplementary material and tables for**

**The Impact of Shanghai's Comprehensive Smoke-Free Legislation on Hospital Admission and Mortality Rates for Chronic Obstructive Pulmonary Disease：An interrupted time series analysis**

[**Appendix : Comprehensive Public Smoking Ban in Shanghai, China**	2](#_Toc191317042)

[**1. Overview of the *Regulations of Shanghai Municipality on Smoking Control in Public Places***	2](#_Toc191317043)

[**2.** **Participants in Tobacco Control**	3](#_Toc191317044)

[**3. Smoking Restrictions in the 2017 Regulation Amendment**	4](#_Toc191317045)

[**4. Supervision and Enforcement of the 2017 Regulation Amendment**	5](#_Toc191317046)

[**5. Penalties under the 2017 Regulation Amendment**	6](#_Toc191317047)

[**Table S1. Degree of Hospital Admission Rate Model Fit by Poisson and Negative Binomial**	8](#_Toc191317048)

[**Table S2. Degree of Mortality Rate Model Fit by Poisson and Negative Binomial**	8](#_Toc191317049)

**Appendix : Comprehensive Public Smoking Ban in Shanghai, China**

**1. Overview of** **the *Regulations of Shanghai Municipality on Smoking Control in Public Places***

The *Regulations of Shanghai Municipality on Smoking Control in Public Places* (hereafter referred to as the Regulations) has been in effect since March 1, 2010. It bans smoking in certain indoor public spaces, workplaces, public transport, and waiting areas for public transport^1^. On November 11, 2016, the Shanghai People’s Congress amended the Regulation for the first time, expanding the ban to include all indoor spaces, thus enforcing a total indoor smoking ban, which took effect on March 1, 2017. A further amendment was made on October 28, 2022, which also includes e-cigarettes under the smoking ban in public areas. As a result, smoking (including e-cigarettes) is now prohibited in all indoor public spaces, workplaces, public transportation, and certain outdoor areas such as schools, hospitals, and cultural sites^2^.

Compared to before the implementation, the atmosphere of tobacco control and the establishment of smoke-free environments in various venues improved after the first revision of the Regulation, with smoking prohibition signs (including no-smoking symbols and reporting hotlines) becoming more standardized^3^. Since the regulation implemented a complete indoor smoking ban, Shanghai has imposed over 2.2 million CNY in fines annually for smoking violations, reaching nearly 16 million CNY by the end of 2023. The violation rate of smoking in designated no-smoking areas dropped from 37.5% before the legislation to 12.3%, with e-cigarette use at 2.2%. Adult smoking rates have decreased steadily, from 26.9% in 2010 to 19.42% in 2023^4^.

In 2022, the enforcement of tobacco control in Shanghai was further strengthened. A total of 301,163 venues were inspected, 469 venues were fined, and 340 individuals were penalized. The fines amounted to 1,648,750 CNY and 26,900 CNY, respectively, with a total of 1,675,650 CNY in penalties. The violation rate of smoking in legally designated no-smoking areas in the city was 12.3%^5^. The concept of "total indoor smoking ban and no smoking in outdoor areas" has become a new health trend among citizens. The idea of building and sharing a smoke-free environment is increasingly ingrained in the city, providing a "Shanghai solution" for innovative urban governance.

**2.** **Participants in Tobacco Control**

Tobacco control requires the joint efforts of the following participants:

a) **Government agencies, public institutions, and their staff** should adhere to tobacco control regulations and take the lead in fulfilling tobacco control obligations.

b) **Shanghai Municipal Center for Health Promotion** should organize relevant departments to strengthen monitoring and evaluation of tobacco control efforts, conduct regular tobacco control inspections, and publish annual reports on the city's tobacco control work.

c) **Relevant administrative departments and industry associations** should incorporate tobacco control into their routine management within their systems and industries.

d) **The health department** should organize smoking behavior intervention programs, establish a hotline, and provide tobacco control consultation services.

e) **Medical institutions** should provide smoking cessation guidance and support to smokers.

f) **Volunteer organizations, social groups, and individuals** should be encouraged to conduct tobacco control public education activities, organize social supervision, assist smokers in quitting, and offer feedback on tobacco control efforts.

g) **Units and individuals** are encouraged to report violations of the regulation through the "12345" citizen hotline or relevant industry regulatory hotlines.

**3. Smoking Restrictions in the 2017 Regulation Amendment**

**Indoor Restrictions**

All indoor public places, indoor workplaces, and public transportation vehicles are designated as smoke-free. In particular, the following categories of places have expanded their smoke-free restrictions:

a) Hotels will implement a comprehensive smoking ban in all indoor areas;
b) Smoking areas will no longer be allowed in restaurants and entertainment venues;
c) Smoking will be prohibited in meeting rooms, dining areas, and shared workplaces in government offices;
d) Smoking rooms will be removed at airports, railway stations, and port terminals.

**Outdoor Restrictions**

The following outdoor areas of public places are smoke-free:

a) Nurseries, kindergartens, primary and secondary schools, youth activity centers, children's welfare institutions, and other public places primarily used by minors;
b) Maternal and child health hospitals and children's hospitals;
c) Sports venues, performance venues, and areas designated for audiences and competitions or performances;
d) Public cultural heritage sites open to the public;
e) Public transportation vehicle waiting areas with large crowds;
f) Other public places as specified by laws, regulations, or rules.

Municipal and district/county governments may designate additional outdoor areas in other public places as permanent or temporary smoke-free zones based on local conditions.

**4. Supervision and Enforcement of the 2017 Regulation Amendment**

The supervision and enforcement of tobacco control work in Shanghai is implemented according to the following provisions:

a) The education administrative department is responsible for supervising and enforcing tobacco control work in schools of all levels and types.
b) The cultural law enforcement agency is responsible for supervising and enforcing tobacco control in cultural, sports, entertainment venues, hotels, and publicly accessible cultural heritage sites.
c) The institutions responsible for airport and railway law enforcement, as well as the transportation administration law enforcement agencies and the operators of metro lines, are responsible for supervising and enforcing tobacco control on public transport and related public places within their respective duties.
d) The food and drug administration (market supervision department) is responsible for supervising and enforcing tobacco control in the foodservice industry.
e) The public security department is responsible for supervising and enforcing tobacco control in internet cafes and other online service venues.
f) The housing and urban-rural construction department is responsible for supervising and enforcing tobacco control in public elevators within property management areas.
g) The health and family planning administrative department is responsible for supervising and enforcing tobacco control in medical institutions of all levels and other public places and workplaces not covered by the above departments.

**5. Penalties under the 2017 Regulation Amendment**

**Tobacco-free Areas**

If an entity violates the provisions of this regulation, the relevant department will order correction within a specified period and impose a fine between 2,000 to 10,000 CNY. In cases of severe violation, the fine can range from 10,000 to 30,000 CNY.

**Individuals**

Any person smoking in a tobacco-free area and refusing to comply with a warning will be ordered to correct their behavior by the relevant department and fined between 50 to 200 CNY.

**References**

1. Shanghai Municipal People's Government. the Regulations of Shanghai Municipality on Smoking Control in Public Places, 2022. Available: https://law.sfj.sh.gov.cn/#/detail?id=63ad099660b224bab98700cb [Accessed October 1, 2024].
2. Shanghai Municipal People's Government. Smoke-free city takes another important step: Ban on e-cigarettes in public places, 2022. Available: https://www.shanghai.gov.cn/nw4411/20221031/2bd0e227b3614f06b7b0cc6b1fe23cd9.html. [Accessed October 1, 2024].
3. HUANG Xin-yuan, WU Jia-yu, ZHANG Zhen, WU Qian, ZHANG Li, YU Deng-lai, GU Chun-hua, WANG Jing, LIN Tao, ZHENG Pin-pin. Implementaton evaluaton of The Revised Shanghai Regulaton on Smoking Control in Public Places over six months in Pudong New Area of Shanghai[J]. Journal of Environmental and Occupational Medicine, 2019, 36(9): 803-812.
4. People's Daily. The adult smoking rate in Shanghai has dropped to 19.4%, 2024. Available: http://sh.people.com.cn/n2/2024/0305/c134768-40765375.html. [Accessed October 1, 2024].
5. Shanghai Municipal People's Government. The adult smoking rate in Shanghai is below 20%，2023. Available:https://www.shanghai.gov.cn/nw4411/20230301/29c080d134324832914905af2af515fa.html[Accessed October 1, 2024].

**Table S1. Degree of Hospital Admission Rate Model Fit by Poisson and Negative Binomial**

|  | Poisson | | Negative Binomial | |
| --- | --- | --- | --- | --- |
| Degree of model fit | AIC | BIC | AIC | BIC |
| Over 35 | 58.65751 | 3457.735 | 18.88019 | -308.4238 |
| 35-64 | 13.20824 | 67.52962 | 14.90784 | -308.6702 |
| Over 65 | 54.33958 | 3132.599 | 18.58202 | -308.359 |

**Table S2. Degree of Mortality Rate Model Fit by Poisson and Negative Binomial**

|  | Poisson | | Negative Binomial | |
| --- | --- | --- | --- | --- |
| Degree of model fit | AIC | BIC | AIC | BIC |
| Over 35 | 22.86684 | 805.9391 | 15.30098 | -307.9146 |
| 35-64 | 6.899604 | -178.5751 | 8.682552 | -304.405 |
| Over 65 | 21.26741 | 684.0286 | 15.22674 | -308.0173 |
